# Histomorphometric and radioimmunoassay studies of the rat endometrium following peanut oil treatment

**Published:** 2011

**Authors:** Venant Tchokonte-Nana, Benjamin Longo-Mbenza

**Affiliations:** School of Medicine, Faculty of Health Sciences, Walter Sisulu University, South Africa.

**Keywords:** Hyperstimulation, Endometrium, Implantation, Progesterone, Peanut oil

## Abstract

**Background:**

The pregnancy rate during in-vitro fertilization (IVF) following progesterone supplement still remains very low at around 20%.

**Objective:**

To investigate the effects of peanut oil itself on the endometrial receptivity, the pregnancy success rate and fertility during the peri-implantation time in hyper stimulated and normal rats.

**Materials and Methods:**

Thirty-six adult Sprague Dawley rats with at least four regular oestrus cycles were randomly divided into 4 groups: two groups were hyper stimulated by human chorionic gonadotropin (hCG) and treated with progesterone or with peanut oil; the two other groups were not hyper stimulated and treated with saline solution or peanut oil. On day 5.5 of pregnancy, the uterine horns were removed and blood was collected for histomorphometric and serum progesterone evaluation. 12 rats were allowed to continue the presumed pregnancy to term. Analysis of variance (ANOVA) and student t-test were used to compare the means of morphometric and radioimmunoassay data between groups. p-values less than 0.05 were considered statistically significant.

**Results:**

The mean values of morphometric parameters and serum progesterone varied significantly between the groups (ANOVA, p<0.0001). The lowest values of progesterone parameters were observed in the hyperstimulated groups that did not deliver pups; both hyperstimulated groups had deleterious luminal epithelium with varying degrees of mucosal projections. There were isolated decidualised zones observed in hyper stimulated peanut oil group, whereas peanut oil group had the highest number of implantation sites and deliveries.

**Conclusion:**

The results show that hype stimulation reduces the endometrial receptivity, while peanut oil increases endometrial receptivity, pregnancy rates and fertility by triggering decidualisation.

## Introduction

There are many reports on the significant relationship between the morphology of the endometrium and the physiology of implantation in both humans (1-3) and laboratory rats (4-11). 

Beneficial impact of normal histological development of the endometrium for a successful In-vitro fertilization (IVF) and embryo transfer (ET) treatment (3, 12) is also well documented.

Peanut oil is often used as a vehicle for progesterone when administered as a hormonal supplement during IVF (1, 2, 13-20). Other studies reported the role of different types of oil on decidual cell reaction (21, 22) and on the reproductive performance in the rat (23, 24). There is little information in the literature on peanut oil- related pregnancy success rate in animal (24). However, there is no data on the effect of peanut oil itself on decidualisation and fertility.

Therefore, the aim of the present study was to investigate the effects of peanut oil itself on the endometrial receptivity, the pregnancy success rate and fertility during the peri-implantation time in the rat. 

## Materials and methods

**Experimental Animals**

In this retrospective data analysis, archives of paraffin blocks of uterine tissues obtained from 36 adult female virgin Sprague- Dawley rats (35 days old and mean weight of 250±30g) were used. The female rats were randomly selected and were placed three to a cage. Their estrus cycles were monitored by taking daily vaginal smears and only rats having at least three regular 4 day estrus cycles prior to the start of the investigation were used. Ethics approval reference number 99-572b was obtained from the Animal Ethics Screening Committee, University of the Witwatersrand.

**Preparation of drug solution**

All drug solutions were prepared fresh immediately prior to treatment and administered by a veterinarian based at the University. Ovarian stimulation was performed by intra-peritoneally injection of 0.1 ml (20 IU) of a prepared solution of 1,000 IU follicle-stimulating hormone (FSH) (Folligon, Intervet, Johannesburg) and 5,000 IU of human chorionic gonadotropic hormone (hCG, Organon, The Netherlands) at mid-day of mid-diestrus and 24 hours later (at mid-day of late diestrus) respectively, under light anaesthesia induced by 5% halothane vaporized in O_2_ to avoid pain. 0.9 ml of peanut oil (commercially obtained and rendered sterile using pasteurisation and apyrogen with lyophilisation) and was mixed with 18 mg (18 IU) of progesterone (P_4_) (Sigma-Aldrich, St. Louis, MO, USA), while warming to about 50°C and vortexing to provide a final concentration of 20 mg/ml (25). 

**Ovarian hyper stimulation - OHS**

The female rats were divided into 4 groups of 9 rats. The female rats in group 1 and group 2 were hyper stimulated while the female rats in group 3 and group 4 were not hyper stimulated. However, rats in group 3 received an intra- peritoneal injection of 0.1 ml saline solution at mid-day of mid-diestrus, and a further injection of saline solution 24 hours later; while rats in group 4 received an intra- peritoneal injection of 0.1 ml peanut oil at mid-day of mid-diestrus, and a further injection of peanut oil (PNO) 24 hours later. 

**Pregnancy test**

The female rats were then placed at late proestrus with proven males. After mating, pregnancy was confirmed by a predominantly leukocytic vaginal smear with an abundance of mucous secretion, and was termed day 0.5 of pregnancy. At mid-day on day 3.5 of pregnancy female rats were treated as follows:

**Treatment**

Group 1: hyper stimulated female rats were treated with a subcutaneous injection of 75 μl (1.5 IU) of progesterone solution at the scruff of the neck (OHS+P_4_).

Group 2: hyper stimulated female rats were treated with a subcutaneous injection of 75 μl of peanut oil at the scruff of the neck (OHS+PNO).

Group 3: non hyper stimulated female rats were given a subcutaneous injection of 75 μl of saline solution at the scruff of the neck.

Group 4: non hyper stimulated female rats were treated with an injection of 75 μl of peanut oil at the scruff of the neck.

On day 5.5 of pregnancy, 12 hyper stimulated non pregnant female rats and 6 pregnant rats from each non hyper stimulated group 3 and group 4 were used for tissue and blood collection. Three rats from each group in the study (n=12) were allowed to continue pregnancy until pups were delivered or abortion occurred (to evaluate fertility). 

**Collection of samples and tissue processing**

A midline abdominal incision was then made in the anesthetized animal and a 1% pontamine blue solution was injected into the inferior vena cava (IVC) for localization of implantation sites (17). The uterine horns were surgically removed and placed on wax plates in Petri dishes containing phosphate buffer solution (pH 7.2-7.3). 

Small pieces of the uterine horns were cut at the probable implantation sites and fixed for 4 hours in Bouin's solution for histology. The rats were euthanized by introducing 200 mg/kg sodium pentobarbitone into the abdominal cavity and the carcasse disposed of by incineration. The fixed uterine tissues were processed in an automatic tissue processor (Leica, TP 1050, Missouri, and USA). 

Care was taken to ensure that the tissues were orientated in the paraffin wax blocks to allow vertical sections through the luminal epithelium to be cut. Ten serial 4μm thick sections per block were obtained from each block using a motorized rotary microtome equipped with a section transfer system- STS (Microm International HM 355S) and rehydrated through a decreasing concentration of alcohols. Sections were then stained with hematoxylin and eosin prior to examination under a light microscope. Blood (5ml) was collected by cardiac puncture for radioimmunoassay (RIA). Blood was centrifuged at 2500 rpm for 20 minutes and collected in Eppendorf tubes.

**Histomorphometric analysis**

A senior histologist (V. Tchokonte-Nana) was blind of the different groups of animals and recorded the histomorphometric data to define endometrial receptivity, slide boxes were viewed under a Zeiss Axio imager A1 microscope (series number 3517001133) equipped with a Fluorescent HBO 100 (Carl Zeiss Vision GmbH, Zepplinstrasse 4, 85399 München- Hallbergmoor, Germany) and a Carl Zeiss black and white camera. The digital images were segmented and the auto measurement software of Axiovision 4.8 and MTB2004 configuration (both Carl Zeiss Vision GmbH) was used.

 A 20x objective was used for the measurement of the mucosal depth; while 40x objectives was used to determine surface epithelial height and glandular epithelial height. Three random measurements per section were made from each animal in all groups using the following parameters:

a) The mucosal depth was measured in mm from the luminal border of the epithelium to the upper margin of the myometrium.

b) The surface epithelial height was measured in μm from the luminal border of the epithelium to the base of the cells.

c) The glandular epithelial height was measured in μm from the luminal border of the gland to the base of the cells.

The evaluation of the results was made by a scientific advisor (B. Longo-Mbenza).

**Radioimmunoassay**

The kit (Diagnostic Product Corporation, Los Angeles, CA) used for the assay was equipped with human serum-based calibrators having progesterone values ranging from 0.1 to 40 mg/mL and neither extraction nor predilution of the samples was required before assay. The serum progesterone and a radiolabeled progesterone preparation competed for binding to an antibody specific for progesterone. The antibody-bound radiolabeled progesterone was separated and the quantity was determined by counting in a gamma spectrometer (AMETEK, Inc, Oak4 Ridge, USA). Results for the unknown were read from a curve prepared by plotting results for a set of known standards. Sera with pre-determined concentrations were included in every assayed for quality control purposes.

**Statistical analysis**

These continuous data were presented as mean± standard error of the mean (±SEM). The differences between all the groups’ means of the continuous variables were tested for significance by student t-test and ANOVA; post-hoc comparisons for all pairs were performed using Tukey-Kramer HSD test. All statistical tests were two-sided tests with a 5% level of significance using the SPSS software for Windows, version 10.0 (SPSS Inc., Chicago, IL, USA).

**Funding support**

No external funding was received for this study.

## Results

**Pregnancy success rate**

There was no pregnancy in the hyper stimulated female rats in group 1 and group 2. However, the frequency of pregnancy (n=9) in group 3 which had non hyper stimulated rats and received saline solution was similar to that observed in group 4 with non- hyper stimulated female rats which received peanut oil.

**Fertility evaluation**

Female pregnant rats (n=3) in each one of the non-hyper stimulated group 3 and group 4 that were allowed to continue with pregnancy delivered pups. The rats in group 4 that received only peanut oil delivered the highest number of pups (n=20), whereas rats in group 3 that received saline solution had the lowest number of pups (n=9). The male: female sex ratio of pups from the peanut oil group was 14:6, and from the saline group was 6:3. 

**Morphological changes and histomorphometry evaluation**

**Figure 1:**
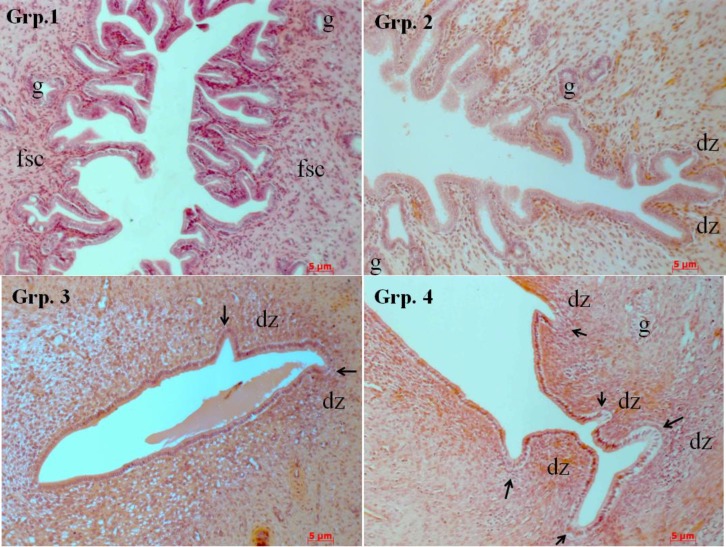
Luminal epithelium of the rat uterus on day 5.5 of pregnancy. H&E photomicrographs of representative uterine sections shown at 20X for hyperstimulated progesterone (group 1), hyperstimulated peanut oil (group 2), control saline (group 3) and control peanut oil (group 4). fsc, fibrobalstic stromal cells; g, glands; dz, decidualized zones, Short arrow, implantation sites

Figure 1 shows histological characterization of implantation, mucosal changes, glandular development and decidualisation of stroma cells in the uterine sections. Implantation site was absent in hyper stimulated rats endometrium treated with combination of progesterone and peanut oil (group 1), as well as in hyper stimulated rats treated with peanut oil only (group 2). 

However, implantation sites were numerous in non-hyper stimulated rats that received peanut oil only (group 4), than in non- hyper stimulated rats that received saline solution (group 3). The luminal epithelial cells in both the hyperstimulated progesterone group and the saline group varied from tall cuboidal to simple columnar. In contrast, the surface epithelial cells in the peanut oil and hyperstimulated peanut oil groups were all tall, simple columnar.

The luminal epithelium of the hyper stimulated rats treated with progesterone showed a marked destruction of the endometrium with very tall projections of the mucosa into the lumen (group 1); whereas moderate mocusal projections were observed in the hyper stimulated rats treated with peanut oil (group 2). In contrast, the luminal surface of the non-hyper stimulated rats treated with saline solution (group 3) and peanut oil only (group 4) were smooth with no mucosal projections.

Decidualised zones were present in the stroma of non-hyper stimulated rats (group 3 and group 4), but sparse in rats hyper stimulated and treated with peanut oil. No decidualised zone was present in hyper stimulated rats treated with progesterone; these rats presented with several fibroblastic cells. However, there was the presence of glandular development in varying number in all the groups.

The mean values of the endometrial mucosal depth, the epithelial height and the glandular height varied significantly between the groups (ANOVA p< 0.0001) (Table I). The lowest values of mucosal depth were observed amongst the rats in the hyper stimulated progesterone group 1, the intermediate value in the hyper stimulated peanut oil group 2, and the highest level in the saline group 3 as well in the peanut oil group 4. Compared with the values in the saline group 3, the values of all the parameters in hyper stimulated peanut oil group 2, surface epithelial height and glandular epithelial height in peanut oil group 4 and, surface epithelial height and mucosal depth in hyper stimulated progesterone group 1 were significantly different, respectively.

Compared with the values in the peanut oil group 4, the values of the mucosal depth and glandular height in the hyper stimulated progesterone group 1 and the values of all the parameters in the hyper stimulated peanut oil group 2 were significantly different, respectively. Compared with the values in the hyper stimulated peanut oil group 2, the values of mucosal depth and surface epithelial height were significantly different respectively. The respective values of parameters in peanut oil group 4 and hyper stimulated peanut oil group 2 were always higher than those compared in other groups.

**Radioimmunoassay**

Table II presents the mean values of serum progesterone which varied significantly across these groups (ANOVA p<0.0001). The serum progesterone levels in the saline group 3 (52.0 ng/mL) and peanut oil group 4 (53.0 ng/mL) were higher than those in the hyperstimulated groups (OHS+P_4_:45.40 ng/mL; OHS+PNO: 45.50 ng/mL). Comparison of means of serum progesterone of each hyperstimulated group with saline group 3 or peanut oil group 4 were statistically significant (p<0.01). 

**Table I:** Mean values of histomorphometric parameters across the groups

		**Luminal epithelium (μm)**		**Glandular height (μm)**		**Mucosal depth (mm)**	
Groups							
	1. Hyper stimulated + progesterone		(n=6) 16.8 ± 0.15^a^^c^		13.0 ± 0.17^b^		0.59 ± 0.01^a^^b^^c^
	2. Hyperstimulated + peanut oil (n=6)		17.7 ± 0.2^a^^b^		13.7 ± 0.12^a^^b^		0.6 ± 0.01^a^^b^
	3. Saline (n=6)		15.3 ± 0.15		13.3 ± 0.13		0.67 ± 0.01
	4. Peanut oil (n=6)		17.0 ± 0.17^a^		14.4 ± 0.16^a^		0.68 ± 0.01
ANOVA p-value		p<0.0001		p<0.0001		p<0.0001	

Statistically significant differences by Student’s t-test:

a : significant with respect to saline group, p< 0.01.

b : significant with respect to peanut oil, p< 0.01.

c : significant with respect to hyper stimulated peanut oil, p< 0.05.

**Table II:** Mean values of serum progesterone across the groups

**Groups**	**Progesterone (ng/ mL)**
1. Hyper stimulated+preogesterone (n=6)	45.40
2. Hyper stimulated+peanut oil (n=6)	45.5
3. Saline (n=6)	52.0
4. Peanut oil (n=6)	53.0
ANOVA p-value	p<0.0001
	

## Discussion

**Histomorphometric analysis**

This study showed a significant difference between non-hyper stimulated group peanut oil only treated group 4 and the rest of the groups with respect to the endometrial mucosal depth, the luminal epithelium height, the glandular epithelial height, the serum progesterone levels during the peri-implantation period, frequency and fertility. 

A study by Critchley *et al* (1) reported morphometrical data showing that using progesterone after ovarian hyper stimulation decreased the height of luminal epithelium, the glandular epithelium and also decreased the stromal thickness. 

These findings demonstrated that epithelium in these hyperstimulated animals was cuboidal. In this study, the epithelial height of the hyperstimulated animals varied from tall cuboidal to simple columnar which is closely similar. The progesterone reduced the height of the luminal and glandular epithelium as well as the decidual reaction. It appears that the increase in values of morphometric parameters and serum progesterone is caused by the effect of the injection of the exogenous peanut oil alone. 

Gidley-Baird *et al* (20) suggested that progesterone levels alone were not inhibitory for implantation in mice and may help protect implantation of the embryo against the inhibitory effects of high estradiol levels. Furthermore, the significant increase in the epithelial height accompanied by higher levels of serum progesterone and highest number of pups delivered in the peanut oil group contradicts previous reports that suggested an increase in the height of epithelial cells impedes implantation (5, 11).

The present study is consistent with previous investigations that reported adverse conditions created by hyper stimulation on the endometrium of women undergoing IVF- ET (1).

The mucosal depth in the hyper stimulated groups was significantly reduced compared to that of non-hyperstimulated groups (saline and peanut oil). When comparing the mucosal depth of the hyperstimulated progesterone group in this study to that study by Kramer *et al* (5) on hyper stimulated rats, it appears that a lower mucosal depth does not support implantation.

Sundstrom (26) suggested that the mucosal depth or thickness could be used as an indication for the success of implantation. Low mucosal depth was reported as possibly a result of the influence of the progesterone: estradiol ratio, which could be suspected in this study. High levels of estradiol are associated with lower mucosal depth and lack of implantation (5, 27). Therefore, Delisle *et al* recommend cautious interpretation of the endometrial measurements in clinical practice (28). 

It was noted, however, that the glandular epithelial height in the peanut oil group was the highest in this study, while the glandular epithelial height was similar in the other groups. This might be an indication of the effect of peanut oil alone on the secretory activity of the endometrium. Also, the appearance of sparse decidualised zones within the stroma of hyper stimulated rats treated with peanut oil in group 2 might be a clear indication of the effects of peanut oil on the endometrium of the female rats. Oil containing antioxidants was reported to have no toxic effects on fertility (23) and general reproductive performance in normal rats (24).

**Radioimmunoassay studies**

Our interpretation of the radioimmunoassay data (serum progesterone) was meaningful of the fact that neither the extraction nor predilution of the samples was done. This may explain the present values out of the reference value range. 

The low level of progesterone in the hyperstimulated rats may stimulate estradiol receptors as emphasized by Guillomot *et al* (19), resulting in increased levels of estradiol, which affects the progesterone: estradiol ratio and thereby may interfere with implantation (5, 7, 10, 11, 29, 31). Similar observations were noted from investigations by Hung *et al* (32), indicating that an imbalanced hormonal state could impede implantation with subsequent reduction of pregnancy rates. 

Considering only the single treatment of progesterone in this study, one might argue that the duration of progesterone treatment in the experimental animals could not restore the progesterone: estradiol ratio required for implantation to occur in this group. The optimal histomorphometric parameters and serum progesterone around the time of implantation may explain the observed high fertility in the peanut oil group and it appears that peanut oil could trigger a decidual reaction around the time of implantation. 

The animals in the hyperstimulated groups did not deliver pups compared with those in the saline group and peanut oil group because of the low levels of serum progesterone and consequently the low mucosal height observed in the hyperstimulated rats. This low level of progesterone impedes implantation (5).

Artificial insemination in women was found to improve the conception rate, but generally does not increase fertility (the number of births per conception). Techniques for increasing fertility in animals by stimulating and controlling ovulation have increased the conception rate. 

The use of progesterone and other hormonal compounds significantly increases the conception rate, but the high cost of hormonal products and the adverse side-effects may prevent their more widespread use. Other compounds, such as peanut oil as used in the present study, have also been used to increase fertility by controlling and stimulating ovulation in animals. 

In an experiment by Juneja *et al* (24), all female mice treated with neem oil (Azadirachta, a known toxic oil) during pre-implantation period had pregnancy block; while mice treated with peanut oil, use as vehicle in control group induced implantation and pregnancy. Although no follow up was done on the offspring in this study, no observed toxicity and abnormalities were noted in the mothers and the pups (23).

**Limitations and criteria for techniques**

Techniques such as RIA may give inaccurate values of serum progesterone. To optimize a high concentration of peanut oil within the hypothalamic-hypophyseal circulation, we injected peanut oil subcutaneously at the scruff of the neck.

## Conclusion

Peanut oil administration without hyper stimulation induced higher and significant endometrial receptivity, increase number of pregnancies and deliveries than progesterone in stimulated rats. Our study indicates the possibility of using a cheap and effective product such as peanut oil as an aid to optimize endometrial development leading to increased fertility in animals.
